# Surface Plasmon Resonance-Induced Stiffening of Silver Nanowires

**DOI:** 10.1038/srep10574

**Published:** 2015-05-29

**Authors:** Xue Ben, Harold S. Park

**Affiliations:** 1 Department of Mechanical Engineering, Boston University, MA 02215 Boston

## Abstract

We report the results of a computational, atomistic electrodynamics study of the effects of electromagnetic waves on the mechanical properties, and specifically the Young’s modulus of silver nanowires. We find that the Young’s modulus of the nanowires is strongly dependent on the optical excitation energy, with a peak enhancement occurring at the localized surface plasmon resonance frequency. When the nanowire is excited at the plasmon resonance frequency, the Young’s modulus is found to increase linearly with increasing nanowire aspect ratio, with a stiffening of nearly 15% for a 2 nm cross section silver nanowire with an aspect ratio of 3.5. Furthermore, our results suggest that this plasmon resonance-induced stiffening is stronger for larger diameter nanowires for a given aspect ratio. Our study demonstrates a novel approach to actively tailoring and enhancing the mechanical properties of metal nanowires.

FCC metal nanostructures such as gold and silver exhibit localized surface plasmon resonance (LSPR), which is a unique optical response that occurs upon interaction with incident electromagnetic waves such as light at specific wavelengths^1^,^2^,^3^,^4^,^5^,^6^ within the visible spectrum. The application areas of LSPR are remarkably broad, and include single molecule sensing and detection^3^,^7^,^8^,^9^, photothermal treatments for cancer^10^,^11^,^12^, optical sensing, tagging and imaging applications^13^,^14^,^15^,^16^, and as a novel approach to enhancing the efficiency of silicon-based thin film photovoltaic devices and solar cells^6^,^17^,^18^.

In addition to their fascinating optical properties, the mechanical properties of metal nanostructures have also been intensely studied in recent years^19^. Many unique behaviors have been reported, which generally arise due to the large surface area to volume ratio that these metal nanostructures exhibit. Specific examples include nanowires that are both ultra strong and ductile^20^,^21^, exhibits near ideal strength^22^,^23^,^24^, surface-stress-induced phase transformations^25^, shape memory and pseudoelasticity^26^,^27^, martensitic phase transformations^28^, and unexpected, surface-mediated mechanisms of plastic deformation^29^,^30^,^31^.

While the fields of nanoplasmonics and nanomechanics are independently large and active, there has to-date been little intersection between the two, though the idea of using mechanical strain to actively tailor and enhance the optical properties of metal nanostructures has been investigated^32^,^33^,^34^,^35^. In particular, what has not been studied is the reverse effect, i.e. whether nanooptical effects such as LSPR can be used to actively tailor and enhance the mechanical properties of metal nanostructures, though we note that this reverse optomechanical effect has been observed in semiconductors by Zhao *et al.*^36^ for ZnO nanobelts, where the ZnO nanobelts exhibited significant elastic stiffening as measured using nanoindentation if illuminated by light with a photon energy that exceeds the 3.34 eV band gap of ZnO.

We report here, by coupling atomistic electrodynamic computational techniques for the optical properties^37^ and standard molecular statics for the mechanical properties^38^,^39^, the strong effect of LSPR on the Young’s modulus of silver nanowires. We demonstrate that the Young’s modulus of silver nanowires is sensitive to the LSPR wavelength, and shows a substantial increase when silver is excited at the LSPR wavelength. The plasmonically-driven enhancement in Young’s modulus is shown to reach nearly 15% for a 2 nm cross section nanowire with an aspect ratio of 3.5. Our work demonstrates for the first time that the elastic properties of metal nanostructures, and in particular the Young’s modulus, can be actively altered and strongly enhanced by optically exciting the electrons of the metal at the localized surface plasmon resonance wavelength.

## Computational Methodology

When an electromagnetic field interacts with a silver nanowire, a frequency-dependent dipolar response is excited in each atom. These induced dipoles result in an optical force that will either augment or oppose any mechanical force that is applied to probe the mechanical properties of the nanostructure^40^,^41^. Furthermore, the polarization-induced optical force that results will likely be at a maximum at the plasmon resonance wavelength, which will thus strongly impact the mechanical properties that are observed in the metal nanostructure through the dipole-induced force coupling between the atoms.

To study this coupled electromechanical problem, we write the frequency (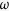)-dependent dynamic electromechanical total energy of the nanostructure as the sum of the mechanical and electrodynamic energies as^40^,^42^


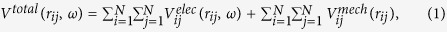


where 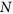 is the total number of atoms in the system and 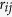 is the distance between atoms 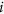 and 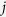. The mechanical potential energy 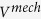, and the resulting interatomic forces for silver is obtained using the well-established embedded atom (EAM) potential^39^, which is known to accurately represent both the bulk and surface properties for transition FCC metals^43^. The boundary value problem represented by Eq. (1) is that of solving for the atomic bond lengths 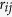 that minimize the total energy 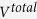 for a nanostructure that exhibits a frequency-dependent response to an externally applied electrodynamic field.

The calculation of the optical force is less standard, so we present it in further detail here. Complete details of the methodology are provided in the Supplemental Materials. We account for these polarization-induced forces using a modification of the recently developed atomistic electrodynamic model of Jensen and Jensen^37^,^44^. In this model, we associate an atomic polarizability with each atom and calculate the induced dipole for each atom self-consistently through their interactions with each other as well as the externally applied electrodynamic field using of classical electrodynamics.

The atomistic electrodynamic model of Jensen and Jensen^37^ is similar to the discrete dipole approximation (DDA)^45^,^46^, but with certain key differences. First, the dipoles are associated with individual atoms in the Jensen and Jensen model, while they are associated with cubical meshing points in the DDA. Second, instead of assuming a uniform dipolar polarizability, a coordination number-dependent polarizability is employed^47^ to better reflect the non-bulk nature of surface, edge and corner atoms.

In the atomistic electrodynamic model, each atom has a frequency-dependent induced atomic dipole 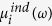, and therefore the electrodynamic total energy 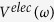 of the nanosystem can be written as


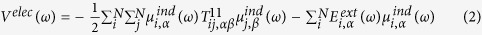



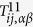
 is the dipole-dipole interaction tensor, which is normalized to eliminate the polarization catastrophe^37^,^48^.

The dipole for each atom is obtained self-consistently by taking the derivative of Eq. (2) with respect to the induced dipole *μ*^ind^(*ω*), giving the following set of linear equations


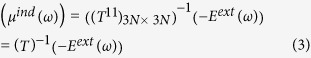


Once the unknown dipole on each atom *μ*^ind^(*ω*) is obtained from Eq. (3), the total electrodynamic energy of the system is written as


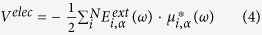


where μ* is the solution of the atomic dipole in Eq. (3). The optical force 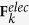 on each atom 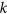 can be obtained by differentiating the total energy in Eq. (4) with respect to the atom positions to yield


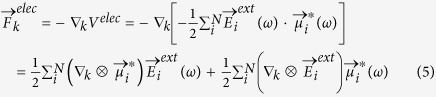


We note that, for the optical force, the dipoles are frequency-dependent, and the force and energy must be written in time-averaged form, which results in an additional factor of 0.5 for both the total energy and force, as compared to the electrostatic case.

A key modification to the atomistic electrodynamic model presented above is to account for discrete nanoscale surface effects, where atoms that lie at corners, surfaces and edges have a different coordination number (i.e. number of bonding neighbors) than do bulk atoms, which will impact their dielectric response and dipolar polarizability. We capture these effects in the present work by adopting the method first proposed by Payton *et al.*^47^, with all details given in the Supplemental Material, including the modifications of the experimentally measured dielectric function of Johnson and Christy^49^.

As discussed above, the optical forces are obtained based on Eq. (5), while the mechanical forces are obtained using the EAM potential for silver^39^. To implement this coupling, the optical forces were implemented in a standalone function that was called and used to augment the mechanical force during each conjugate gradient iteration performed by the open source LAMMPS^50^ atomistic simulation code.

The simulations were performed as follows. First, silver nanowires of various sizes and aspect ratios were created with the atoms placed at the bulk lattice spacing of 4.09 Å. The nanowires were then relaxed to their equilibrium configurations under the competing influences of mechanical surface stresses^51^, which cause the nanowire to contract in order to increase the coordination number of surface atoms^25^,^26^, and the optical forces, which cause the nanowires to expand due to dipole-dipole repulsion. The resulting equilibrium configuration is always at a state of compression, i.e. the nanowire length is shorter than when initially created at the bulk lattice positions, though it is important to note that the compressive strain is smaller than for the purely mechanical case, i.e. when compression due to surface stresses occurs.

Once the equilibrium configuration was found, the nanowires were deformed uniaxially in compression by applying a compressive displacement that scaled from zero at one end to a maximum value at the other end while the optical force was still applied, while again allowing the nanowire to find the resulting equilibrium configuration. At equilibrium for each configuration during the compression process, the atoms that are not at the boundary experience zero net forces, while the fixed atoms at the left and right ends of the nanowire experience nonzero forces that are needed to keep them at the fixed positions. The total force that is needed to compress the nanowire can be obtained by summing over all the nonzero forces at the fixed end. The Young’s modulus of the nanowire was calculated by extracting the reaction force at the displaced end, converting it to stress by normalizing by the nanowire cross sectional area, and calculating the slope of the resulting stress versus strain curve.

## Effects of LSPR on Nanowire Young’s Modulus

We first characterize the optical field effect on a specific silver nanowire with fixed geometric dimensions to elucidate the effects of electric field intensity on its mechanical properties. Specifically, we consider a 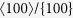 nanowire with dimensions 4 × 2 × 2 nm^3^, subject to optical fields with peak electric field intensity ranging from 0.1 to 0.3 V/Å. In the following discussion, we note that all electric field intensities refer to the peak field intensity.

**Figure 1:**
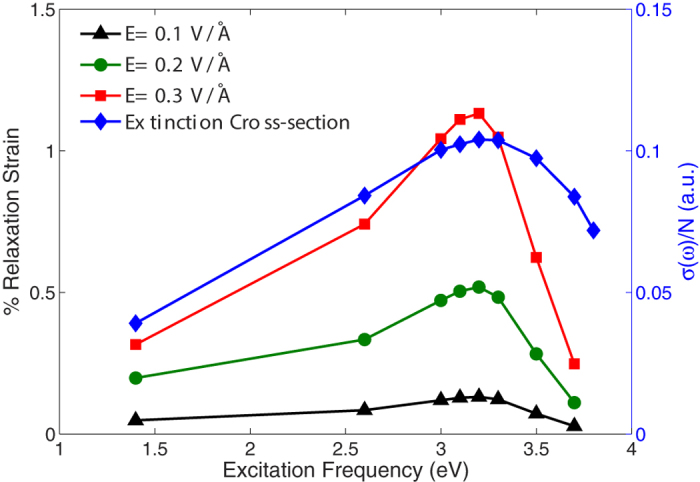
Percent change in length, or relaxation strain, as a function of optical field frequency and intensity for a 4 × 2 × 2 nm^3^ silver nanowire. Corresponding extinction spectrum is overlaid to emphasize connection between relaxation strain and the surface plasmon resonance frequency.

Due to the optical field excitation, repulsive dipolar forces are generated among the atoms, with the repulsion being strongest at the nanowire axial surfaces. Therefore, as shown in Fig. (1), the nanowire length increases as compared to relaxed nanowires without electrodynamic excitation before any external compression is applied, where the relaxation strain is defined as 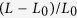, where 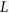 is the deformed nanowire length and 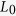 is the length of mechanically relaxed nanowire, i.e. after contraction due to surface stresses. Again, we note that the overall state of strain in the nanowires is compressive, but the relaxation strain in Fig. (1), and all subsequent figures, is positive due to the fact that the dipolar repulsion generated by the optical field causes the nanowire to elongate from the mechanically relaxed configuration resulting from the intrinsic mechanical surface stresses^25^,^26^,^27^.

It is also interesting to examine the relaxation strain spectrum in Fig. (1). In particular, because silver is a dispersive material, its strong frequency-dependent optical response results in a strongly frequency-dependent initial relaxation strain. Therefore, we have overlaid in Fig. (1) the extinction cross section for the 4 × 2 × 2 nm^3^ nanowire. As can be seen, the maximum relaxation strain is observed to occur at the resonance peak for the silver nanowire, which occurs at around 3.2 eV for the 4 × 2 × 2 nm^3^ nanowire shown in Fig. (1). We also note that the relaxation strain increases with increasing electric field intensity, though the frequency at which the relaxation strain is a maximum is independent of the electric field intensity.

The dependence of the relaxation strain on the excitation frequency is because localized surface plasmon resonance is a manifestation of the collective oscillations of the electrons in the nanowire due to the incoming electrodynamic field. As a result of this resonance, atoms experience the strongest polarization in response to the external field at the resonant frequency. At resonance, the dipolar repulsion is strongest, which manifests itself through a maximum in the repulsive optical force, and thus a maximum in the positive relaxation strain in Fig. (1) caused by the repulsive dipolar force.

**Figure 2:**
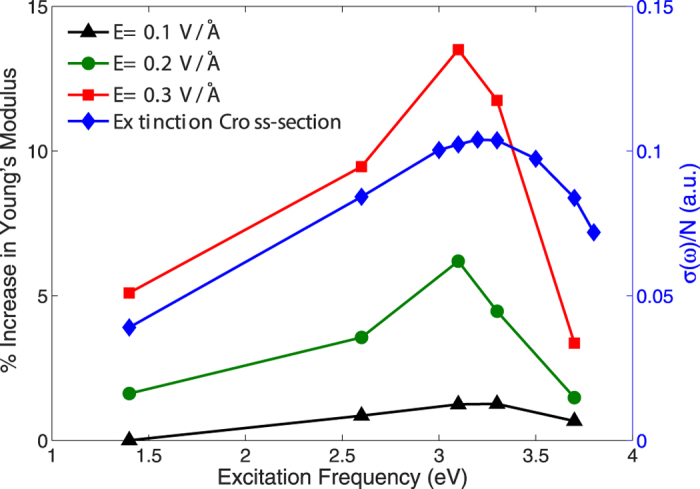
Percent change in the Young’s modulus as a function of optical field frequency and intensity for a 4 × 2 × 2 nm^3^ silver nanowire. Corresponding extinction spectrum is overlaid to emphasize connection between the Young’s modulus enhancement and the surface plasmon resonance frequency.

Having established the relaxation strain as a function of optical frequency and intensity, we now show in Fig. (2) the percent change in Young’s modulus for the same 4 × 2 × 2 nm^3^ silver nanowire. The percent change in Young’s modulus is calculated as 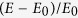, where 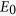 is the Young’s modulus of the nanowire when it is not subject to any applied electromagnetic radiation, and 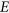 is the Young’s modulus for the nanowire subject to the prescribed optical frequency and intensity. We note that the normalizing value 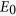 is size-dependent due to nonlinear elasticity arising from contraction due to tensile surface stresses, and follows previously established trends^52^. As can be seen, there is a great similarity between the relaxation strain trend in Fig. (1) and the Young’s modulus in Fig. (2), where the maximum enhancement in the Young’s modulus occurs at the plasmon resonance frequency of 3.2 eV, where again we have overlaid the extinction spectrum to highlight the connection between the optical and mechanical properties. Furthermore, the Young’s modulus enhancement increases with increasing electric field intensity, reaching about 14% for an electric field of 0.3 V/Å.

The increase in the Young’s modulus when the nanowire is subject to externally applied electrodynamic fields is due to the initial relaxation strain, which because it is positive (or less negative than the purely mechanical case due to surface stresses) ensures that the nonlinear elastic softening^52^, which occurs due to the large compressive relaxation strains that the 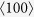 nanowires undergo in the absence of any externally applie fields, is alleviated in these nanowires. Because Fig. (1) shows that the resonance strain is frequency-dependent, this offers substantial flexibility in tuning the mechanical stiffness of the nanowires, as shown in Fig. (2).

**Figure 3:**
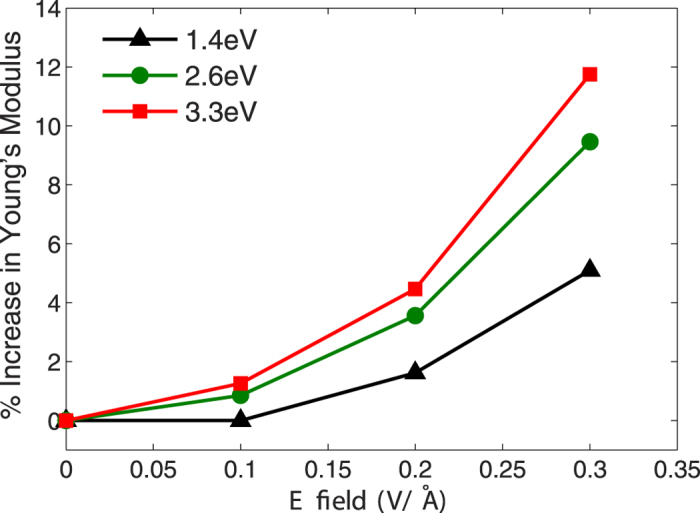
Percent change in Young’s modulus as a function of optical field frequency and intensity for a 4 × 2 × 2 nm^3^ silver nanowire.

Another issue to quantify concerns the change in Young’s modulus as a function of optical frequency and intensity. While we previously established in Fig. (2) that the Young’s modulus enhancement is electric field-dependent, we wish to quantify the impact of the optical frequency. We therefore show in Fig. (3) the change in Young’s modulus for different electric field intensities at different optical frequencies. As can be seen, the change in Young’s modulus is nonlinear for not only the near-resonant frequency of 3.3 eV, but also for the off resonance frequencies of 1.4 and 2.6 eV. However, it is clear that the increase in Young’s modulus is strongly dependent on the excitation frequency, and the closer the frequency to the LSPR frequency, the greater the change in stiffness. Therefore, in Fig. (3), the curve at 3.3 eV shows the largest enhancement in Young’s modulus with increasing electric field intensity.

While we have considered only one nanowire size up until now, it is well-known that the optical properties of metal nanostructures are strongly size and shape-dependent^4^,^53^,^54^,^55^. We now consider both of these factors and how they influence the plasmonically-driven enhancement in the Young’s modulus for silver nanowires.

**Figure 4:**
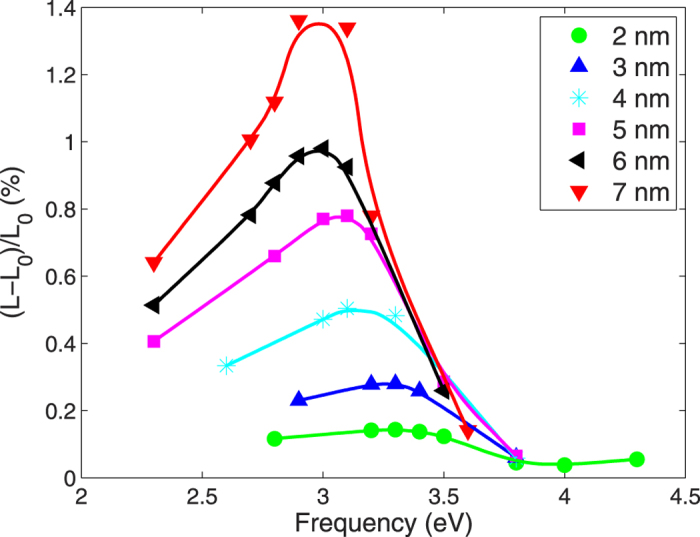
Relaxation strain for nanowires with cross sectional size of 2 nm and different axial lengths ranging from 2 to 7 nm under an electric field intensity of 0.2 V/Å.

Figure (4) shows the relaxation strain for nanowires with a constant cross sectional size of 2 nm, but for axial lengths ranging from 2 to 7 nm subject to an electric field intensity of 0.2 V/Å. As can be seen, a steady red shift is observed in the relaxation length spectrum with increasing length, which is to be expected as the resonance relaxation strain is caused by LSPR, and because for different size nanowires, the restoring force experienced by the electrons inside the nanowire changes, which modifies the resonance frequency. In addition, the value of the relaxation strain increases with increasing aspect ratio, reaching a maximum of nearly 1.4% tension for the 7 × 2 × 2 nm^3^ nanowire.

**Figure 5:**
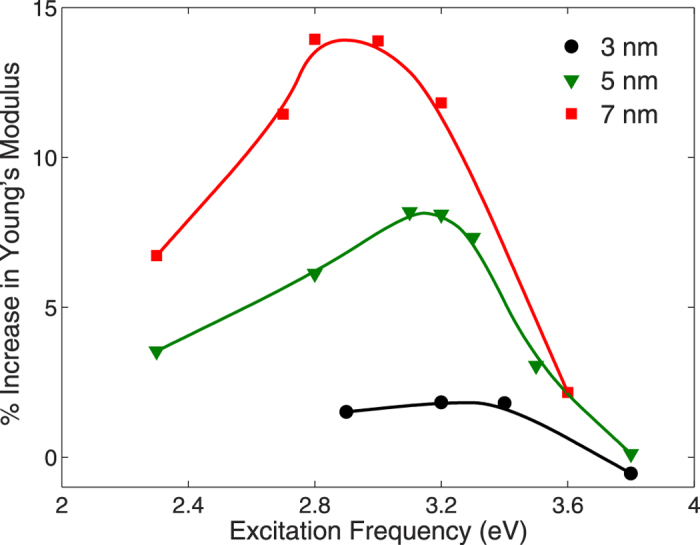
Percent change in Young’s modulus for nanowires with fixed cross sectional length of 2 nm, and axial lengths of 3, 5 and 7 nm subject to an electric field intensity of 0.2 V/Å.

Figure (5) shows the percent change in Young’s modulus for nanowires with a constant cross sectional size of 2 nm, with axial lengths of 3, 5 and 7 nm under an electric field intensity of 0.2 V/Å. Similar to the relaxation strain in Fig. (4), a red shift in the frequency at which the maximum enhancement in the Young’s modulus occurs is observed. Furthermore, the enhancement in the Young’s modulus increases with increasing aspect ratio, with a 14% enhancement seen for the 7 × 2 × 2 nm^3^ nanowire. These results are in contrast to recent experimental studies using time-resolved transient extinction measurements to study the Young’s modulus of plasmonic nano particles^56^, where no variation in the Young’s modulus with respect to the bulk value was found. This is because that the underlying operant mechanism for the ultrasmall nanowires considered in the present work lies in the nonlinear elastic softening effects due to the large compressive strains induced by surface stresses, is not active for the experimentally-considered nanowires due to the fact that nanowires with diameters around 30 nm undergo very little surface-stress-induced compressive strains.

**Figure 6:**
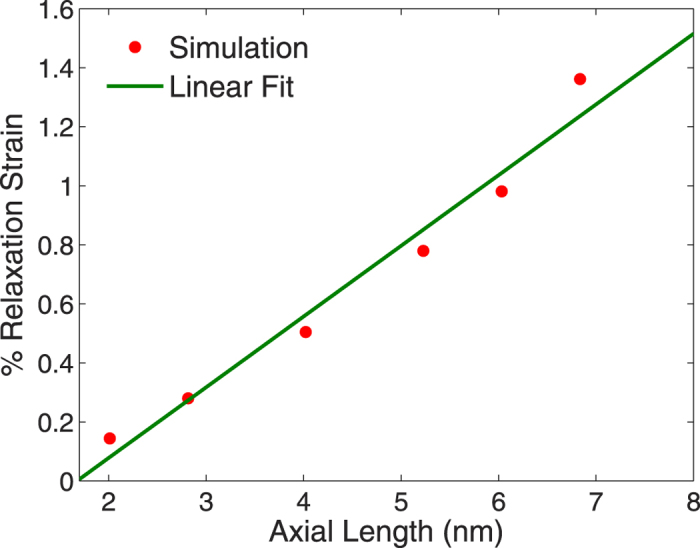
Relaxation strain for 2 nm cross section silver nanowires with increasing axial length, or aspect ratio. The relaxation strains are those at the resonance peak, and were calculated for an electric field of 0.2 V/Å.

**Figure 7:**
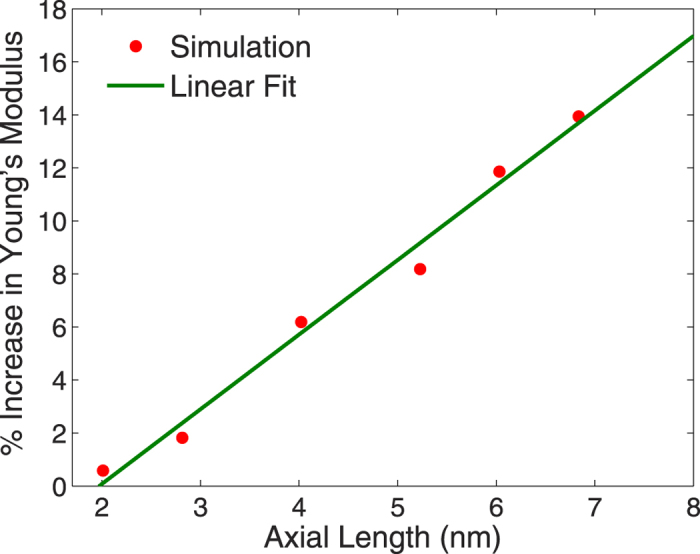
Percent change in Young’s modulus for 2 nm cross section silver nanowires with increasing axial length, or aspect ratio. The values for Young’s modulus are those at the resonance peak, and were calculated for an electric field of 0.2 V/Å.

We plot the relaxation strain as well as the Young’s modulus enhancement as a function of nanowire aspect ratio, both for an applied electric field of 0.2 V/Å, in Figs. (6) and (7), where both are calculated at the resonance frequency of the nanowire. As can be seen, as the nanowire length increases, the optical tensile forces become stronger and thus both the relaxation strain and the Young’s modulus enhancement become increasingly positive. The Young’s modulus, in particular could be enhanced by more than 20% when the aspect ratio increase above about 5.

It is also interesting to note that the linear increase seen in Figs. (6) and (7) are similar to the linear increase in redshift of the plasmon resonance wavelength with aspect ratio previously observed for metal nanostructures^53^,^57^,^58^. For the linear variation in relaxation strain shown in Fig. (6), we note that it is also found in calculations where the excitation is from an electrostatic, and not electrodynamic field^59^. Thus, this interesting effect is not due to the surface plasmon resonance, but instead appears to result from the electric field-induced dipolar interactions.

**Figure 8:**
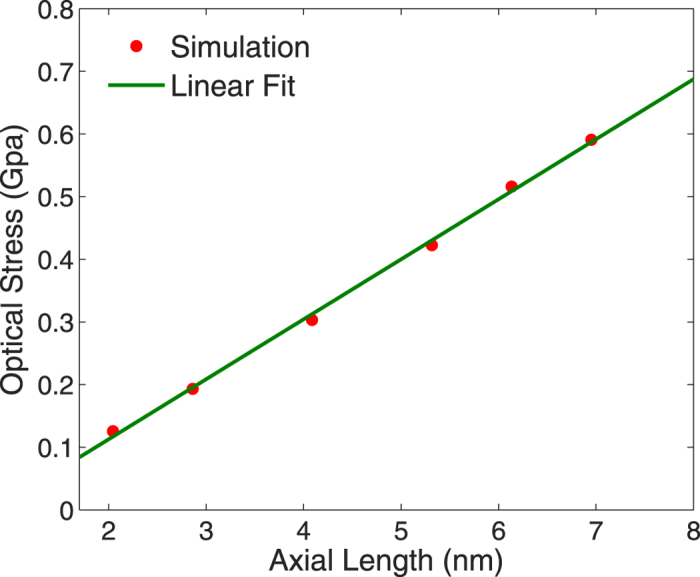
Variation in the optical stress with increasing axial length, or aspect ratio, for a silver nanowire with cross sectional length of 2 nm.

However, it is also known that longer nanowires with larger aspect ratios exhibit larger relaxation strains due to surface stresses because of their larger ratio of transverse to total surface area^60^, which is why we find increasing relaxation strains for nanowires with larger aspect ratios. Furthermore, we calculate the optical stress, or the stress due to the applied electromagnetic radiation, for nanowires with increasing axial length or aspect ratio. As shown in Fig. (8), the stress due to the optical excitation also exhibits a linear scaling with respect to aspect ratio, which explains the linear dependency in the relaxation strain and Young’s modulus seen in Figs. (6) and (7).

**Figure 9:**
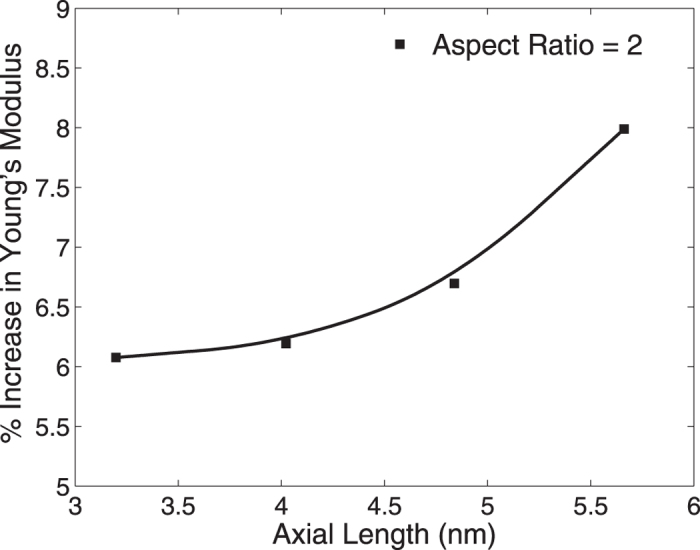
Percent change in Young’s modulus for silver nanowires with fixed aspect ratio of 2.

The last issue we address is that of the size effect, i.e. to determine whether this LSPR-induced stiffening will be enhanced or reduced for larger nanowires. Due to computational limitations, we considered nanowires with a constant aspect ratio of two, but with cross sectional lengths ranging from about 3 to 5.5 nm. As shown in Fig. (9), the Young’s modulus increases with increasing axial length, and thus cross sectional length. This result helps in our understanding of the competing factors that govern the change in the Young’s modulus with increasing axial and cross sectional length, under the external field excitation. As previously shown by Park and Klein^60^, the relaxation strain of the nanowires when no electric field is applied increases with increasing axial length, for a fixed cross sectional size, and decreases with increasing cross sectional size for a constant length. A consequences of the increase in compressive strain due to surface stresses is nonlinear elastic softening (reduction in the Young’s modulus) for 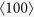 nanowires as compared to bulk silver, as shown by Liang *et al*^52^.

However, when the nanowires are excited by the incident dynamic field, the compressive strain due to surface stresses is reduced due to the repulsive dipolar interactions, which also reduces the nonlinear elastic softening due to the compressive strain, which finally results in enhanced stiffness of the nanowires. Nanowires with increasing axial length (for a fixed cross sectional size) exhibit a stronger reduction of the compressive strain, which results in a larger change in the Young’s modulus. In contrast, nanowires with increasing cross sectional length (for a fixed axial length) exhibit a weaker reduction of the compressive strain, and thus a smaller change in the Young’s modulus, as also shown in our previous work^59^. Thus, though all nanowires become stiffer as compared to the purely mechanical case, the geometry of the nanowire governs the magnitude of the stiffness increase.

For a fixed aspect ratio, at least for the small sizes considered in the present work, the results in Fig. (9) show that when the axial and cross sectional lengths increase with the same proportion, the percentage change in Young’s modulus still goes up for the larger sizes (both increasing axial and cross sectional lengths). This implies that it is more effective to make the nanowire stiffer by increasing its axial length than by decreasing the cross sectional size.

## Conclusions

In conclusion, we have utilized a coupled atomistic, electromechanical formulation to demonstrate that localized surface plasmon resonance can be utilized to significantly enhance the mechanical stiffness of silver nanowires. The Young’s modulus enhancement was found to have a linear dependence on the aspect ratio, and can be larger than 20% for silver nanowires with aspect ratios larger than 5. Finally, the utilization of optical excitation enables substantial flexibility in actively tailoring and enhancing the mechanical properties of metal nanostructures due to the fact that the stiffness enhancements are strongly frequency dependent.

## Additional Information

**How to cite this article**: Ben, X. and Park, H. S. Surface Plasmon Resonance-Induced Stiffening of Silver Nanowires. *Sci. Rep.* **5**, 10574; doi: 10.1038/srep10574 (2015).
